# The psoriasis-protective TYK2 I684S variant impairs IL-12 stimulated pSTAT4 response in skin-homing CD4+ and CD8+ memory T-cells

**DOI:** 10.1038/s41598-018-25282-2

**Published:** 2018-05-04

**Authors:** C. Enerbäck, C. Sandin, S. Lambert, M. Zawistowski, P. E. Stuart, D. Verma, L. C. Tsoi, R. P. Nair, A. Johnston, J. T. Elder

**Affiliations:** 1 Department of Dermatology, University of Michigan, Ann Arbor, MI USA; 2 Ingrid Asp Psoriasis Research Center, Department of Dermatology, Linköping University, Linköping, Sweden; 3 Department of Biostatistics, University of Michigan, Ann Arbor, MI USA; 4 Ann Arbor Veterans Affairs Health System, Ann Arbor, MI USA

## Abstract

Tyrosine kinase 2 (TYK2) belongs to the Janus kinase (JAK) family of tyrosine kinases, which transmit signals from activated cytokine receptors. GWAS have consistently implicated *TYK2* in psoriasis susceptibility. We performed an in-depth association analysis of *TYK2* using GWAS and resequencing data. Strong genetic association of three nonsynonymous variants in the exonic regions of the *TYK2* gene (rs34536443, rs12720356, and rs2304256) were found. rs12720356 encoding I684S is predicted to be deleterious based on its location in the pseudokinase domain. We analyzed PBMCs from 29 individuals representing the haplotypes containing each of the significantly associated signals. STAT4 phosphorylation was evaluated by phospho-flow cytometry after CD3/CD28 activation of cells followed by IL-12 stimulation. Individuals carrying the protective I684S variant manifested significantly reduced p-STAT4 levels in CD4 + CD25 + CD45RO+ (mean Stimulation Index (S.I.) 48.08, n = 10) and CD8 + CD25 + CD45RO + cells (S.I. 55.71, n = 10), compared to controls homozygous for the ancestral haplotype (S.I. 68.19, n = 10 (p = 0.002) and 76.76 n = 10 (p = 0.0008) respectively). Reduced p-STAT4 levels were also observed in skin-homing, cutaneous lymphocyte associated antigen (CLA)-positive CD4 and CD8 cells from I684S carriers. No significant changes in p-STAT4 for the psoriasis-associated variant rs34536443 was found. These data establish the functional significance of the *TYK2* I684S variant in psoriasis susceptibility.

## Introduction

Psoriasis is an immunologically mediated inflammatory disease that affects about 1–2% of the population of the Western world^1^. Psoriasis is characterized by intense hyperproliferation and disturbed maturation of the epidermal cells, inflammatory infiltrates of the dermis and epidermis, and extensive new blood vessel formation^2^. Genetic results support the role of a dysregulated immune system through pathways that involve cells of both innate and acquired immunity^3^. Genome-wide association studies (GWAS) have identified 63 independent susceptibility loci in European-origin individuals, most of which appear to be regulatory in nature^4^. The major challenge of the post-GWAS era is the identification of causal variants and genes, which may be more straightforward for coding than for regulatory variation.

Tyrosine kinase 2 (TYK2) belongs to the Janus kinase (JAK) family of tyrosine kinases, which transmit signals from activated cytokine receptors^5^. GWAS identified *TYK2* as a psoriasis susceptibility gene in 2010^6^, this finding has since then been replicated by others, including ourselves^7,8^. In addition, the locus exhibits large effect size, and is one of the few psoriasis susceptibility regions whose strongest genetic signals involve coding variants. Genetic variation in *TYK2* has also been associated with several other autoimmune diseases, including multiple sclerosis, systemic sclerosis, Type 1 diabetes, Crohn’s disease, ulcerative colitis, and systemic lupus erythematosus^9–17^. TYK2 associates with the cytoplasmic domain of cytokine receptors to transmit cytokine signals by phosphorylating receptor subunits^18,19^. In a recent study, selective inhibitors for TYK2 were used to determine the specific contribution of TYK2 catalytic activity to cytokine responses, using a receptor-distal phospho-STAT readout^20^. TYK2 catalytic activity was found to be required for signaling events downstream of IL-12 and IL-23, but did not contribute significantly to signaling downstream of IFN-α, IL-6, IL-10, and IL-22, which instead depended on JAK1 catalytic activity^20^. Interestingly, JAK2 contributed to IL-23, but not to IL-12 signaling, suggesting that IL-12 might be the most appropriate stimulus for the evaluation of *TYK2* coding variation. In this study, we therefore selected IL-12 as a stimulus for the evaluation of functional effects of genetic variation in *TYK2*.

## Results and Discussion

### The identification of three nonsynonymous variants in *TYK2* using GWAS and resequencing data

**Figure 1:**
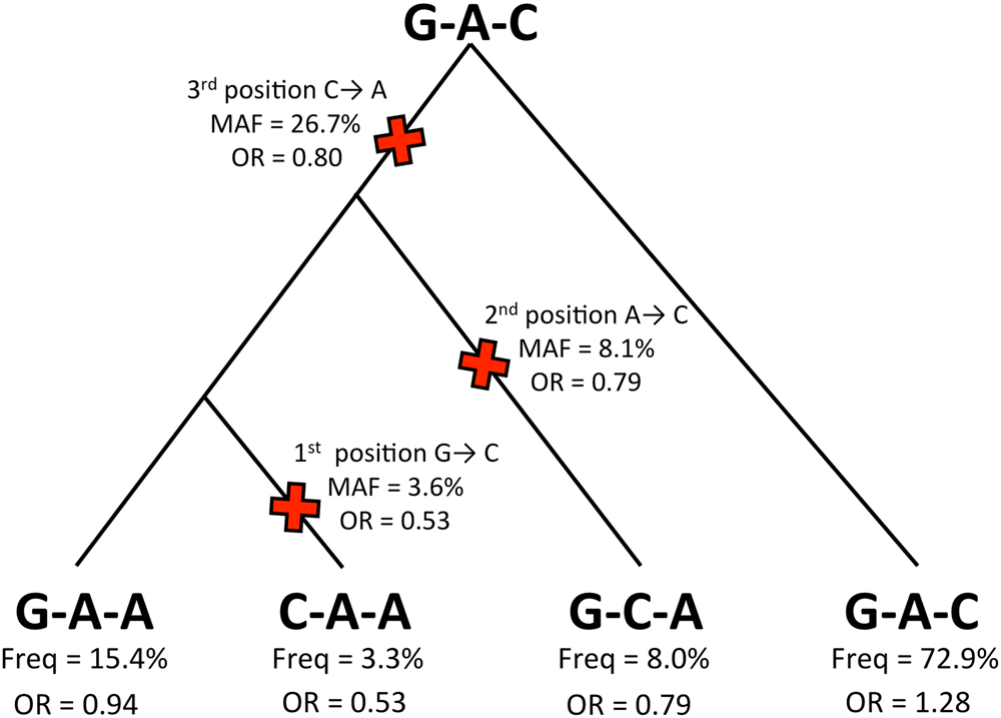
TYK2 variants and haplotypes analyzed in this study. The figure shows the most parsimonious ancestry for the four most common TYK2 haplotypes (frequency > 0.5%). The TYK2 gene tree is rooted at the ancestral G-A-C haplotype with red “X”’s indicating the most parsimonious history of mutation events giving rise to the three independently associated psoriasis SNPs rs34536443 G > C, rs12720356 A > C, and rs2304256 C > A. The ancestral allele at each SNP was determined using the UCSC Genome Browser and in each case agreed with the major allele. The three SNPs are presented in chromosomal (p → q) order on the plus strand, which corresponds to C-terminal to N-terminal order in the *TYK2* gene. The minor/protective allele frequency (MAF) and single marker odds ratio (OR) are shown for each SNP. Frequencies and odds ratios are also shown for each haplotype.

Using Plink version 1.07^21^ to perform conditional haplotype-based association analysis of the *TYK2* genomic region (Supplementary Information and Table S1), we found strong independent genetic associations between psoriasis and three nonsynonymous variants in exonic regions of the *TYK2* gene (rs34536443 G > C, rs12720356 A > C, and rs2304256 C > A) (Fig. 1). We found that the minor (i.e., derived) allele for each SNP was psoriasis-protective, with the ancestral haplotype conferring the highest risk. Notably, among haplotypes with at least 1% frequency in the underlying population, the protective alleles at the 1^st^ (rs34536443) and 2^nd^ (rs12720356) positions occur only with the protective allele at the 3^rd^ position (rs2304256) **(**Fig. 1**)**. SNP rs34536443 encoding TYK2 P1104A is located in the kinase domain, is conserved across 20 mammalian species, predicted to be strongly damaging by Polyphen^22^ and was found to alter TYK2 phosphorylation in response to IFN-β^23^. SNP rs12720356 encoding TYK2 I684S is located in the pseudokinase domain, is also conserved across 20 mammalian species, predicted to be strongly damaging, and was found to be catalytically-impaired yet signaling-competent in the context of interferon (IFN)-α activation, likely due to its ability to exert a heterodimeric scaffolding function with other JAKs^24^. SNP rs2304256 encoding V362F resides in the FERM domain involved in cytokine receptor binding, is not predicted to be damaging, and is not conserved across 20 mammalian species or across other JAK family members^25^.

### The *TYK2* I684S variant impairs IL-12 stimulated pSTAT4 response

Based on the aforementioned pharmacological evidence for specificity of TYK2 signaling downstream of the IL-12 receptor^20^, we used phospho-flow cytometry to measure IL-12-stimulated T-cell STAT4 phosphorylation in CD3/CD28-activated peripheral blood mononuclear cells (PBMC). In preliminary experiments, we determined the optimal time and dose of IL-12 stimulation (Figure S1) as well as CD3/CD28 activation (Figure S2). As shown in Figure S3, IL-12 receptors are induced in response to CD3/CD28 activation in purified T-cell populations, to a very similar extent to that seen in PBMC. We studied individuals representing three rs34536443-rs12720356-rs2304256 haplotypes: GAC (ancestral, n = 10), CAA, n = 9, and GCA, n = 10. Characteristics of the study population are shown in Table S2. In our study, the CAA and GCA haplotypes were each paired with the ancestral GAC haplotype. The GAA haplotype, whose component SNP rs2304256 C > A is not highly conserved or predicted to be damaging, was not evaluated.

**Figure 2:**
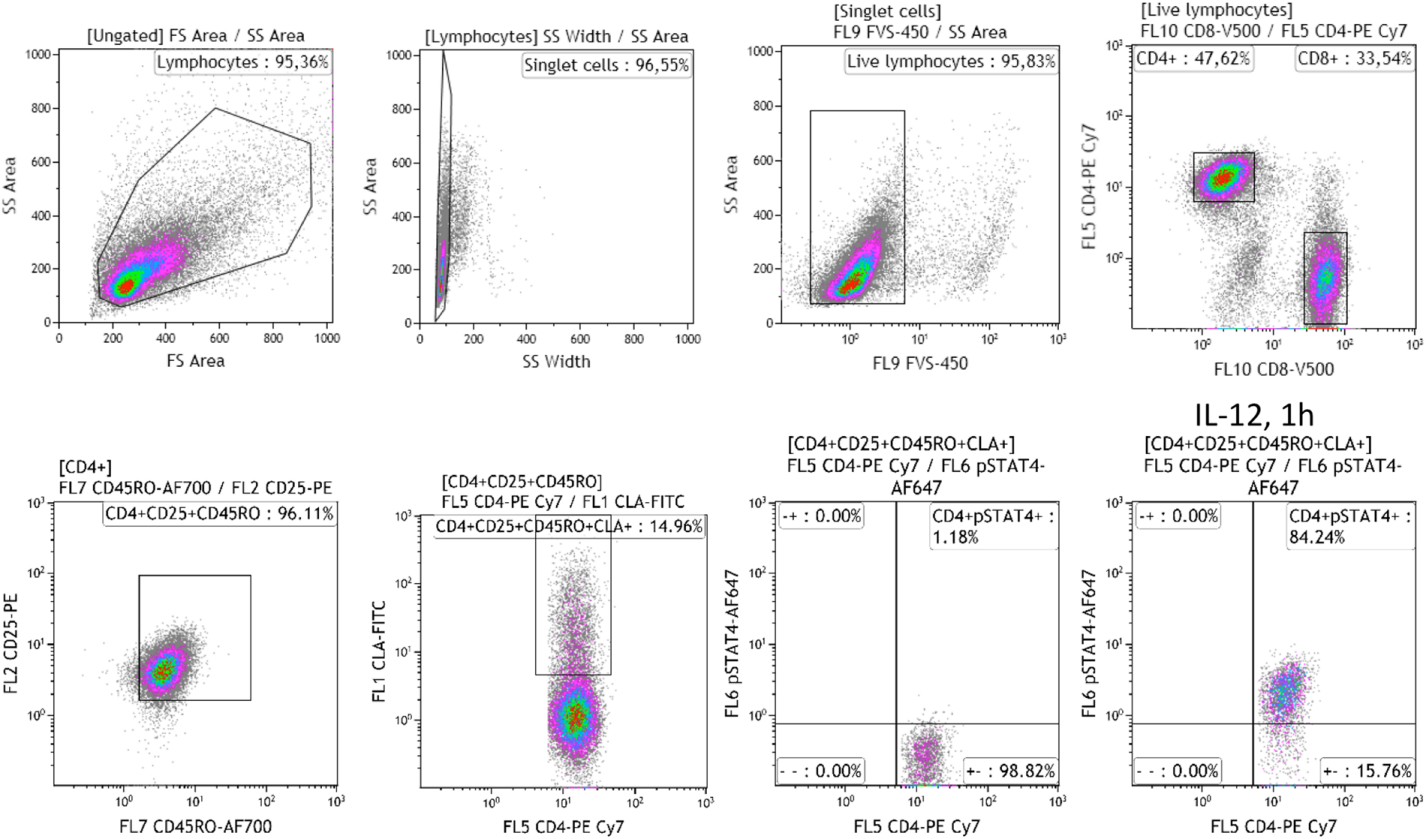
Gating strategy for phospho-flow cytometry of p-STAT4. Lymphocytes were gated on a forward (FS) and side scatter (SS) density plot and on a SS area versus SS width density plot to remove doublet cells. A live/dead stain (FVS-450) was used to exclude dead lymphocytes. Live, activated, memory, skin-homing CD4+ or CD8+ T lymphocytes were selected based on the expression of CD25, CD45RO, and cutaneous lymphocyte antigen (CLA). pSTAT4 expression was detected in cells activated by anti-CD3/CD28 and stimulated with or without 50 ng/ml IL-12 for 1 h.

**Figure 3:**
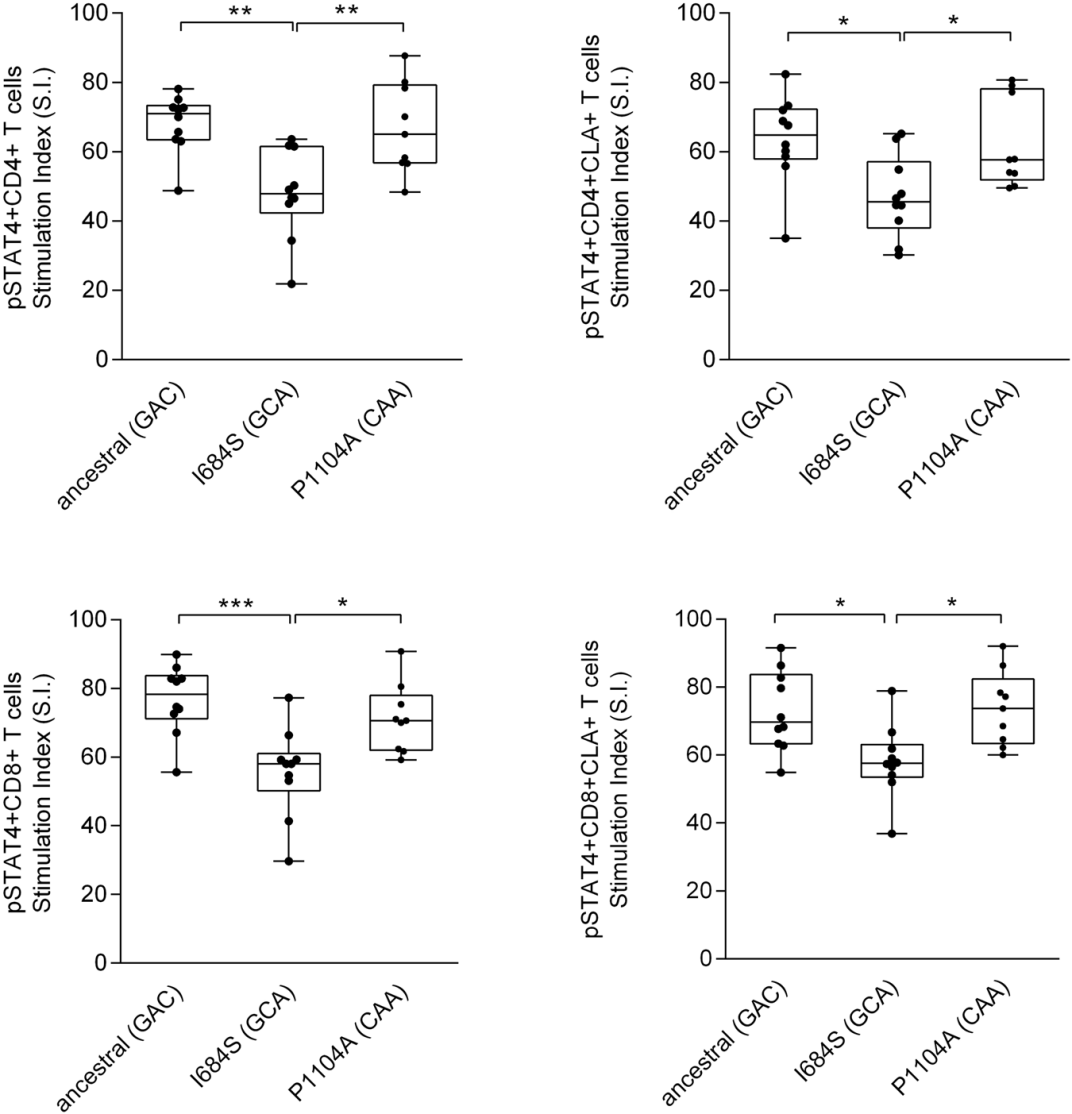
Functional effects of genetic variants in *TYK2* on STAT4 phosphorylation in CD4+ and CD8+ T lymphocytes after stimulation with IL-12. PBMCs derived from individuals carrying the ancestral haplotype (n = 10) and from two haplotypes representing the genetic variants I684S (n = 10) and P1104A (n = 9), were cultured for 72 h with anti-CD3/CD28 beads, rested overnight, then stimulated with 50 ng/ml IL-12 for 1 h and analyzed for intracellular pSTAT4 expression by multicolor flow cytometry. Stimulation Index (S.I.) of pSTAT4 in CD25 + CD45RO + CD4+ and in lymphocyte antigen positive (CLA+) CD25 + CD45RO + CD4 + T-lymphocytes (upper panel). S.I. of pSTAT4 in CD25+CD45RO+CD8 and in CLA+CD25+CD45RO+CD8 T- lymphocytes (lower panel). Box plots: midline indicates median, box extends from 25^th^ to 75^th^ percentile, and whiskers indicate minimum to maximum. A *p* value < 0.05 was considered to be significant. *Indicates p < 0.05, ** indicates p < 0.01, and *** indicates p < 0.001.

The gating strategy is shown in Fig. 2. We found that individuals carrying one copy of the GCA haplotype encoding the protective I684S variant (genotype GCA/GAC) had significantly reduced p-STAT4 levels in CD4+ and CD8+, activated (CD25+) memory (CD45RO+) T-cells, compared to individuals homozygous for the ancestral GAC haplotype (Fig. 3). Similar reductions in p-STAT4 were also observed in skin-homing, cutaneous lymphocyte associated antigen (CLA)-positive CD4 and CD8 cells from carriers of the protective I684S variant (Fig. 3**)**. In contrast, we found no significant changes in p-STAT4 for individuals of the CAA/GAC haplotype harboring the psoriasis-associated variant rs34536443 encoding P1104A in the kinase domain, relative to homozygotes for the ancestral haplotype GAC (Fig. 3). We obtained the same result after restricting the analysis to controls (individuals without psoriasis, Figure S4).

Because JAK-family tyrosine phosphorylation is regarded as an obligate downstream signal transduction response to cytokine stimulation, we also assessed TYK2 tyrosine phosphorylation by ELISA. The characteristics of the analyzed individuals are shown in Table S3. However, as shown in Figure S5, there were no significant differences in TYK2 phosphorylation across any of the three haplotypes.

### TYK2 regulates IL-12 dependent gene expression

In order to ask whether any particular gene responses to IL-12 might be specific targets of *TYK2* genetic variation, we carried out RNA-seq analysis of CD3/CD28-activated PBMC harvested after 1 and 4 h of IL-12 treatment. For these experiments, at least five individuals of each haplotype were selected for analysis (Table S4). Differential expression analysis across all three haplotypes as a function of IL-12 treatment identified 52 significantly (FDR <= 0.1 and fold change (FC) log_2_|FC| ≥ log_2_|1.5|) dysregulated genes at 1 h (43 up, 9 down, Table S5) and 233 dysregulated genes at 4 h (132 up, 101 down; Table S6). These dysregulated genes displayed significant overlap with a list of 28 IL-12 responsive genes identified from the literature by searching the NCI Biosystems database (4 and 6 genes at 1 h and 4 h, p = 1.05 × 10^−7^ and 1.09 × 10^−7^, respectively, Tables S5 and S6). Not unexpectedly, genes significantly up-regulated by 4 h of IL-12 treatment were most highly enriched for the Gene Ontology term “Regulation of interferon gamma mediated signaling pathway” (22.8-fold, FDR = 3.9 × 10^−4^, Table S7). Notably, after 4 hours of IL-12 treatment, the most significantly up-regulated gene was IL18RAP (average 2.37-fold, FDR p = 1.0 × 10^−13^), which also manifested a nominally significant effect of *TYK2* genotype (ANOVA p = 7.2 × 10^−3^, Table S10). IL18RAP encodes an accessory subunit of the heterodimeric receptor for interleukin 18 (IL-18), a proinflammatory cytokine involved in inducing cell-mediated immunity. This protein enhances the IL-18-binding activity of the IL-18 receptor and plays a role in signaling by IL-18. IL18RAP is overexpressed 3.4-fold in lesional psoriatic vs. normal skin^26^, and mutations in this gene are associated with susceptibility to Crohn’s disease, celiac disease, and leprosy.

Regarding the functional effects of rs12720356 encoding TYK2 I684S, we have recently shown that the combined effects of IFN-γ and TNF contribute to increased vascular inflammation, a major cause of cardiovascular comorbidity in psoriasis^27^. While we were unable to demonstrate a significant effect of *TYK2* genotype on IFN-γ mRNA expression in this relatively small RNA-seq study (Tables S9 and S10), our finding that IL-12 stimulation rapidly and significantly increases IFN-γ gene expression in anti-CD3/CD28-stimulated PBMC (Tables S5 and S6) has potential functional relevance to systemic inflammation in psoriasis. More generally, a Gene Ontology analysis of genes meeting an ANOVA p-value criterion of 0.05 for an effect of s12720356 genotype demonstrated significant (FDR ≤ 0.1) functional enrichment for “lymphocyte activation” and “T-cell activation” (Table S11). Finally, in support for functional relevance in the skin, we assessed the possible expression quantitative trait locus (eQTL) properties of rs12720356 encoding I684S on our RNA-seq-based gene expression dataset of skin samples^26,28^ (Table S12). The top hit in this eQTL analysis was *GCSAM* (germinal center associated signaling and motility, aka *GCET2*, Gene ID: 257144, eQTL p = 2.3 × 10^−5^), which manifests strongly biased expression not only in lymph nodes, but also in skin (ref. Entrez Gene). Collectively, these suggestive findings merit further analysis in a larger sample.

In this study, we selected individuals for functional studies based on 3-SNP haplotype-based genotypes instead of single-SNP genotypes. As shown in Fig. 1, rs34536443C is found on only one haplotype (CAA), whereas rs34536443G can belong to two different haplotypes—the ancestral GAC haplotype or the derived GAA haplotype—which carry different odds ratios in our study. More importantly, we utilized heterozygotes for the minor alleles at rs34536443 and rs12720356, rather than homozygotes. It is possible that by analyzing heterozygotes, we may have been able to capture an inhibitory effect of the TYK2 684S variant on its “wild-type” 684I counterpart, in the context of TYK2 homodimers bound to the IL-12 receptor. The existence of such homodimers in T-cells is strongly suggested by pharmacological evidence^20^. Our major findings complement those of Dendrou *et al*.^29^, who found a significant reduction in STAT4 phosphorylation in CD4 and CD8 memory T-cells in rs34536443 C/C vs. G/G homozygotes^29^. There were several differences between the two studies with regards to the experimental protocol, which might account for the differences in results. We stimulated whole PBMC, whereas Dendrou *et al*. stimulated purified CD3+ T-cells. We utilized CD3/CD28 beads for 72 hr followed by 1 hr of IL-12 stimulation, whereas Dendrou *et al*. activated with phytohemaglutinin for 72 hr followed by IL-2 for 24 hr prior to stimulation with IL-12 for 15 min. It is worth noting that in both our studies and those of Dendrou *et al*.^29^, hypofunctional TYK2 alleles, which are predicted to be functionally damaging to TYK2 signaling, are associated with a protective effect against psoriasis.

In conclusion, our results demonstrate a significant decrease in STAT4 phosphorylation in GCA/GAC heterozygotes (bearing one copy of the rare C allele at rs12720356 encoding TYK2 684 S in the pseudokinase domain), relative to either GAC/GAC homozygotes or CAA/GAC heterozygotes. This decrease in STAT4 phosphorylation was even detectable in skin-homing, cutaneous lymphocyte associated antigen (CLA)-positive CD4 and CD8 T-cells from carriers of the protective I684S variant. Taken together, these data support the functional significance of the TYK2 I684S variant in psoriasis susceptibility, and set the stage for further mechanistic investigation.

## Methods

### CD3/CD28 activation and IL-12 stimulation of PBMCs

Characteristics of the subjects chosen for analysis of STAT4 phosphorylation are shown in Table S2. Peripheral blood mononuclear cells (PBMC) were obtained by Ficoll-Hypaque centrifugation and cryopreserved in liquid nitrogen until analysis. Cryopreserved PBMCs were thawed, washed by centrifugation at 250 × *g* in complete medium, and activated with 25 µl per 10^6^ cells anti-CD3/anti-CD28 beads (Gibco) for 3 days. Cells were seeded at a density of 1.0 × 10^6^ cells/well (24 well plate) in RPMI 1640 supplemented with 1% L-glutamine, 1% penicillin and streptomycin, and 10% FBS. On the third day, beads were separated from cells by pipetting up and down five times followed by immediate magnetic capture of the beads. The activated PBMCs were rested overnight in fresh medium and then stimulated by addition of 50 ng/ml IL-12 (R&D Systems) for 1 h.

### Flow cytometric analysis of phosphorylated STAT4

After washing, cells were stained with antibodies directed to cutaneous lymphocyte antigen (CLA)-FITC (BioLegend), the activation marker CD25-PE (BD Biosciences), CD3-PerCP-Cy5.5, and CD8-V500 (BD Biosciences), in PBS supplemented with 0.1% FBS and 2% pooled heat-inactivated (HI) human serum for the blocking of Fc-receptors. Cells were then treated with Fixation Buffer (BD Biosciences), and subsequently with Perm Buffer III (BD Biosciences) according to the manufacturer’s protocol. For intracellular detection of pSTAT4, cells were treated with titrated anti-pSTAT4 Alexa Fluor 647 (BD Biosciences) after fixation and permeabilization. Flow cytometry was performed on a Gallios analyser (Beckman Coulter) and data were analyzed using Kaluza® Flow Analysis Software, version 1.3 (Beckman Coulter). Lymphocytes were gated on a forward-(FS) and side scatter (SS) density plot and on a SS area versus SS width density plot to remove cell doublets. A live/dead stain (FVS-450) was used to exclude dead lymphocytes. Live, activated (CD25+) memory (CD45RO+) CD4+ or CD8+ T lymphocytes were selected based on the expression of CD25 and CD45RO, and skin-homing T-cells were further selected based on surface expression of cutaneous lymphoid antigen (CLA). The gating strategy is depicted in Fig. 2. The Stimulation Index (S.I.) was obtained by dividing the percentage of pSTAT4+ cells after IL-12 stimulation by the percentage of pSTAT4+ cells in unstimulated cells. The gate defining pSTAT4 positivity was set such that 1% of IL-12-unstimulated cells fell within the pSTAT4 gate.

### Statistical analysis

We used two-way ANOVA followed by Holm-Sidak test for multiple comparisons for the analysis of pTYK2 measured by ELISA using GraphPad Prism 6. One-way ANOVA followed by Holm-Sidak test for multiple comparisons was used to evaluate the comparison of pSTAT4-upregulation after IL-12 stimulation in individuals carrying nonsynonymous variants or the ancestral haplotype, using GraphPad Prism 6. The results are shown as mean and the standard deviation (mean ± SD). A *p* value < 0.05 was considered to be significant.

Additional methodologic details are provided in the Supplementary Information.

### Database submission

The RNA-seq data described in this work has been deposited in the Gene Expression Omnibus database (GEO accession number TBD).

## Electronic supplementary material

Supplementary information

Supplementary Dataset 1
