# Supplementary material for: The psoriasis-protective TYK2 I684S variant impairs IL-12 stimulated pSTAT4 response in skin-homing CD4+ and CD8+ memory T-cells
Source: Sci Rep. 2018 May 4;8:7043. doi: 10.1038/s41598-018-25282-2 (PMC5935702; doi:10.1038/s41598-018-25282-2)
Supplement: Supplementary file 1 — Supplementary information [file 41598_2018_25282_MOESM1_ESM.docx]

Supplementary Information For:

The psoriasis-protective TYK2 I684S variant impairs IL-12 stimulated pSTAT4 response in skin-homing CD4+ and CD8+ memory T-cells

Charlotta Enerbäck^1, 2^, Charlotta Sandin^2^, Sylviane Lambert^1^, Matthew Zawistowski^3^, Philip E. Stuart^1^, Deepti Verma^2^, Lam C. Tsoi^1, 3^, Rajan P. Nair^1^, Andrew Johnston^1^, and James T. Elder^1, 4^

^1^ Department of Dermatology, University of Michigan, Ann Arbor, MI, USA

^2^ Ingrid Asp Psoriasis Research Center, Department of Dermatology, Linköping University, Linköping, Sweden

^3^ Department of Biostatistics, University of Michigan, Ann Arbor, MI, USA

^4^ Ann Arbor Veterans Affairs Health System, Ann Arbor, MI, USA

Corresponding Author:

Charlotta Enerbäck

Ingrid Asp Psoriasis Research Center, Department of Clinical and Experimental Medicine, Linköping University

SE-581 85 Linköping, Sweden

Phone: +46 10 103 7429

E-mail: charlotta.enerback@liu.se

Running Title: TYK2 I684S variant impairs IL-12 signaling

Key words: psoriasis, immunology, genetics, TYK2

**Supplementary Methods:**

**Study sample and genotyping**

Individuals of Caucasian descent were identified based on TYK2 genotypes obtained from genome-wide [^1^](#_ENREF_1) or Immunochip-based [^2^](#_ENREF_2) association studies of psoriasis. All human subjects provided written informed consent and were enrolled according to the protocols approved by the institutional review board of the University of Michigan Medical School, in adherence with the Declaration of Helsinki principles.

**Haplotyping and association testing**

Haplotype phasing and conditional haplotype-based association testing were carried out using version 1.07 of Plink [^3^](#_ENREF_3) for a sample of 11,675 unrelated European-origin individuals that included all individuals who participated in this study. This sample was genotyped on an Affymetrix Axiom Biobank Genotyping Array that included the three *TYK2* SNPs of this study among its exome content, along with a GWAS backbone [^4^](#_ENREF_4) (**Table S1**). The framework of the association analysis was modeled after the approach of Purcell et al. [^5^](#_ENREF_5). The accuracy of the haplotypes determined by Plink was verified with version 2.2 of SHAPEIT [^6^](#_ENREF_6), which was applied to the three *TYK2* SNPs of this study, along with 2,875 additional genotyped markers within a 5 Mb window centered on *TYK2*. Conservation was assessed and ancestral alleles were determined using the 20 Mammals Conservation track of the UCSC Genome Browser. For all three SNPs, the common allele was the human reference allele. For rs34536443 and rs12720356, the reference alleles were the same for human and all 20 mammalian species. For rs2304256, the human reference allele agreed with chimp, bonobo, gorilla, and orangutan, but diverged for gorilla and the remaining primate and mammalian species.

**ELISA detection of phosphorylated TYK2**

Approximately 0.6 x10^6^ cells from IL-12 stimulated cultures and control cultures were analyzed for pTYK-detection. The cells were lysed with Bio-Plex™ Cell Lysis Kit (Bio-Rad) according to the manufacturer’s protocol. Cell lysates were collected and stored at -80°C for further analysis. The lysates were thawed at RT and then placed on ice. Detection of pTYK2 was performed according to the manufacturer’s protocol by using pTYK2 ELISA (Sigma catalog # RAB1006).

**RNA purification and RNA-Seq**

Approximately 0.6 x10^6^ cells from PBMC cultured for 72h with CD3/CD28 beads and further treated with or without 50 ng/ml IL-12 for 24h were pelleted and 350µl RLT Plus buffer (Qiagen) was added. After vortexing for 30 seconds, the solution was stored at -80°C for further analysis. Total RNA was purified by using the Qiagen RNEasy Mini Prep kit (Qiagen, Germantown, MD) following manufacturer’s instructions. Massively-parallel 50 nucleotide single-end cDNA sequencing (RNA-seq) was performed on an Illumina HiSeq 4000 instrument using libraries prepared with the NEBNext Ultra RNA Library Prep Kit with the Poly-A selection module for isolating mRNA. RNA-seq reads were mapped to the human genome build 37 by using Tophat2 [^7^](#_ENREF_7), and gene expression was quantified by HT-Seq [^8^](#_ENREF_8). Normalization and differential expression analysis were conducted using DESeq2 [^9^](#_ENREF_9). IL-12-responsive genes were identified by searching the NCBI BioSystems database. Twenty-eight IL-12 responsive genes were identified, and for 11 of them, we found publications in PubMed supporting them as responsive to IL-12 signaling mediated by STAT4.

**Functional annotation and eQTL analysis**

Statistical significance of overlap between gene lists (**Tables S5 and S6**) and Gene Ontogeny functional enrichment categories **(Tables S7, S8 and S11**) was assessed using the hypergeometric test [^10^](#_ENREF_10). For eQTL analysis, we associated the genotypes of the variant with the expression values for all skin-expressed transcripts using genotyped RNA-seq samples from our psoriasis cohort whose skin biopsies were subjected to RNA-seq [^10^](#_ENREF_10)^,^[^11^](#_ENREF_11). The normalized expression level was further inverse normalized to ensure robustness of the eQTL association analysis, and we employed a linear regression model to test the effect of rs12720356 after using the top two principal components computed using the skin (psoriasis and normal)-specific transcriptome as covariates.

**Supplementary Tables**

**Table S1.** Single marker and haplotype main and conditional effect tests of association for three TYK2 SNPs.

|  | *rs34536443* | *rs12720356* | *rs2304256* |  | HF | OR_OM_ | OR_HS_ | P_HS (logit)_ | P_HS (score)_ | P_SV-H_ |
| --- | --- | --- | --- | --- | --- | --- | --- | --- | --- | --- |
|  |  |  |  |  |  |  |  |  |  |  |
| *H*_1_ | **G** | C | A |  | 0.080 | -ref- | 0.788 | 1.0 x 10^-6^ | 8.4 × 10^-7^ | 1.2 x 10^-20^ |
| *H*_2_ | C | **A** | A |  | 0.033 | 0.689 | 0.527 | 9.9 x 10^-17^ | 6.6 x 10^-16^ | 7.7 x 10^-11^ |
| *H*_3_ | **G** | **A** | A |  | 0.154 | 1.212 | 0.939 | 0.089 | 0.071 | 3.6 x 10^-25^ |
| *H*_4_ | **G** | **A** | **C** |  | 0.729 | 1.364 | 1.276 | 3.1 x 10^-16^ | 9.8 x 10^-15^ | 2.5 x 10^-11^ |
|  |  |  |  |  |  |  |  |  |  |  |
| MAF | 0.036 | 0.081 | 0.267 |  |  |  |  |  |  |  |
| OR_SS_ | 0.530 | 0.792 | 0.798 |  |  |  |  |  |  |  |
| P_SS_ | 1.1 x 10^-18^ | 1.3 x 10^-6^ | 2.9 x 10^-14^ |  |  |  |  |  |  |  |
| P_IE_ | 1.6 x 10^-11^ | 6.7 x 10^-4^ | 0.0014 |  |  |  |  |  |  |  |
| P_SV-S_ | 7.7 x 10^-11^ | 5.6 x 10^-19^ | 2.5 x 10^-11^ |  |  |  |  |  |  |  |

*H*_1_-*H*_4_ are haplotypes; *rs34536443, rs12720356* and *rs2304256* are biallelic markers (risk allele for each marker is bolded red and larger font); MAF is minor allele frequency for markers; OR_ss_ is allelic odds ratios of the minor (protective) allele under single marker tests; P_SS_ is single marker allelic p-value; P_IE_ is independent effect test p-value; P_SV-S_ is SNP-based sole-variant test p-value; HF is haplotype frequency; OR_OM_ is haplotypic odds ratio under the ominibus test; OR_HS_ is haplotypic odds ratio under haplotype-specific tests using logistic regression; P_HS (logit)_ is haplotype-specific test p-value using logistic regression; P_HS (score)_ is haplotype-specific test p-value using an allelic score (contingency) test; P_SV-H_ is haplotype-based sole-variant test p-value. Ominibus test P_logit_ = 7.0e-025; ominibus test P_score_ = 1.9e-022.

**Please Note:**  Tables S2-S8 are presented as spreadsheets in a separate Excel workbook

**Table S2.** Characteristics of the study sample used for phospho-flow cytometry of pSTAT4.

**Table S3.** Characteristics of the study sample used for phospho-TYK2 ELISA.

**Table S4.** Characteristics of the study sample used for RNA-seq.

**Table S5.** Genes with significantly (FDR < 0.1) altered expression in IL-12-activated, CD3/CD28-stimulated PBMC after 1 hr of IL-12 treatment. A list of 28 IL-12-regulated genes identified from the literature by searching the NCI Biosystems database is shown in the rightmost column. Genes from our dataset that overlap with the 28 genes identified from the literature are highlighted in yellow.

**Table S6.** Genes with significantly (FDR < 0.1) altered expression in IL-12-activated, CD3/CD28-stimulated PBMC after 4 hr of IL-12 treatment. A list of 28 IL-12-regulated genes identified from the literature by searching the NCI Biosystems database is shown in the rightmost column. Genes from our dataset that overlap with the 28 genes identified from the literature are highlighted in yellow.

**Table S7.** Gene Ontology term enrichment for genes up-regulated by 4h of IL-12 treatment.

**Table S8.** Gene Ontology term enrichment for genes down-regulated by 4h of IL-12 treatment.

**Table S9.** Genes manifesting an effect of *TYK2* genotype after 1 hr of IL-12 treatment. The effect of *TYK2* genotype is assessed by ANOVA. Red highlighting: ANOVA p < 0.05. Yellow highlighting: fold change (FC) maximum likelihood estimate ≥ 1.55 or ≤ 0.67. Green highlighting: FDR-adjusted p value for fold change ≤ 0.1. FC data are same as for the DEGs shown in Table S5, except that all genes meeting QC criteria are included in this table.

**Table S10.** Genes manifesting an effect of *TYK2* genotype after 4 hr of IL-12 treatment. The effect of *TYK2* genotype is assessed by ANOVA. Red highlighting: ANOVA p < 0.05. Yellow highlighting: FC maximum likeihood estimate ≥ 1.55 or ≤ 0.67. Green highlighting: FDR-adjusted p value for fold change ≤ 0.1. FC data are same as for the DEGs shown in Table S5, except that all genes meeting QC criteria are included in this table.

**Table S11.** Functional enrichment for genes manifesting an effect of *TYK2* genotype after 4 hr of IL-12 treatment.

**Table S12**. eQTLs for SNP rs12720356 in psoriatic skin (data from Li et al., Jnvest Dermatol 134: 1828-38, 2014). Please note that the approved name for GCET2 is now GCSAM.

**Supplementary Figures and Legends**

**
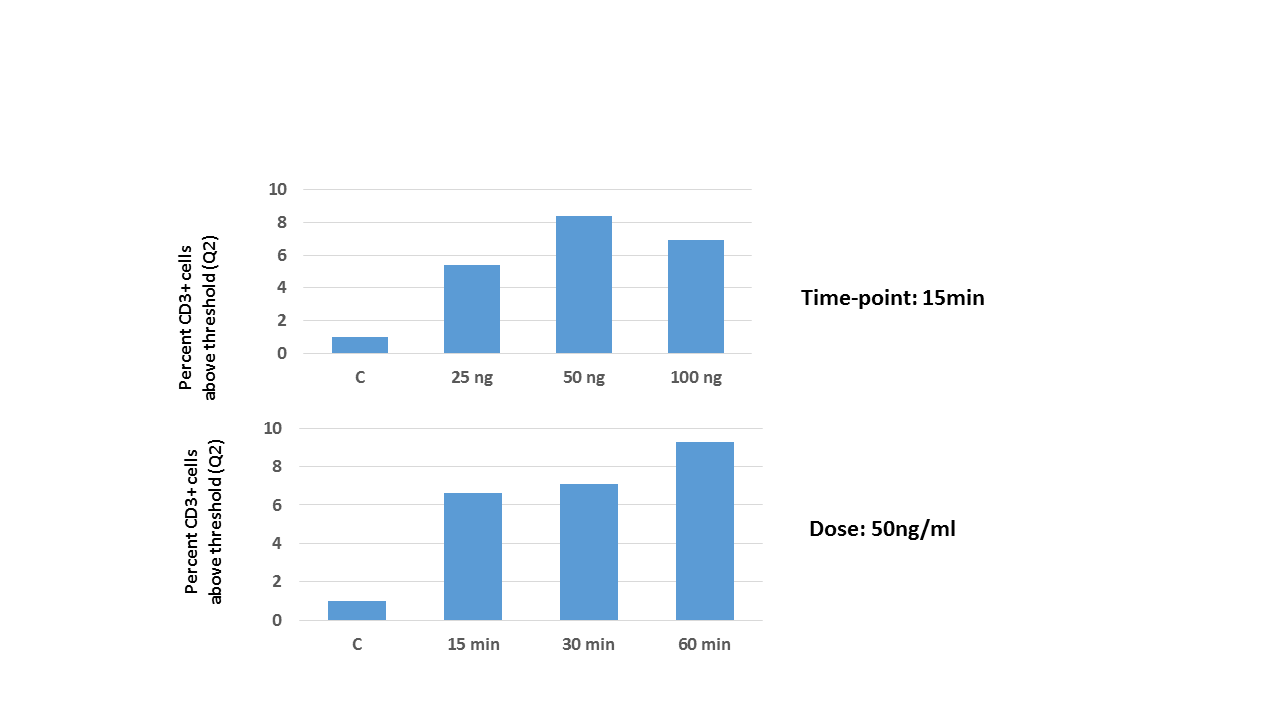
**

**Figure S1: Selection of dose and time point for IL-12 stimulation**

Column-purified T-cells were stimulated with anti-CD3/CD28 beads for 72h, stimulated with various doses IL-12 (25 ng/ml, 50 ng/ml, or 100 ng/ml) for 15, 30, or 60 min, then fixed, permeabilized, and stained intracellularly for pSTAT4.


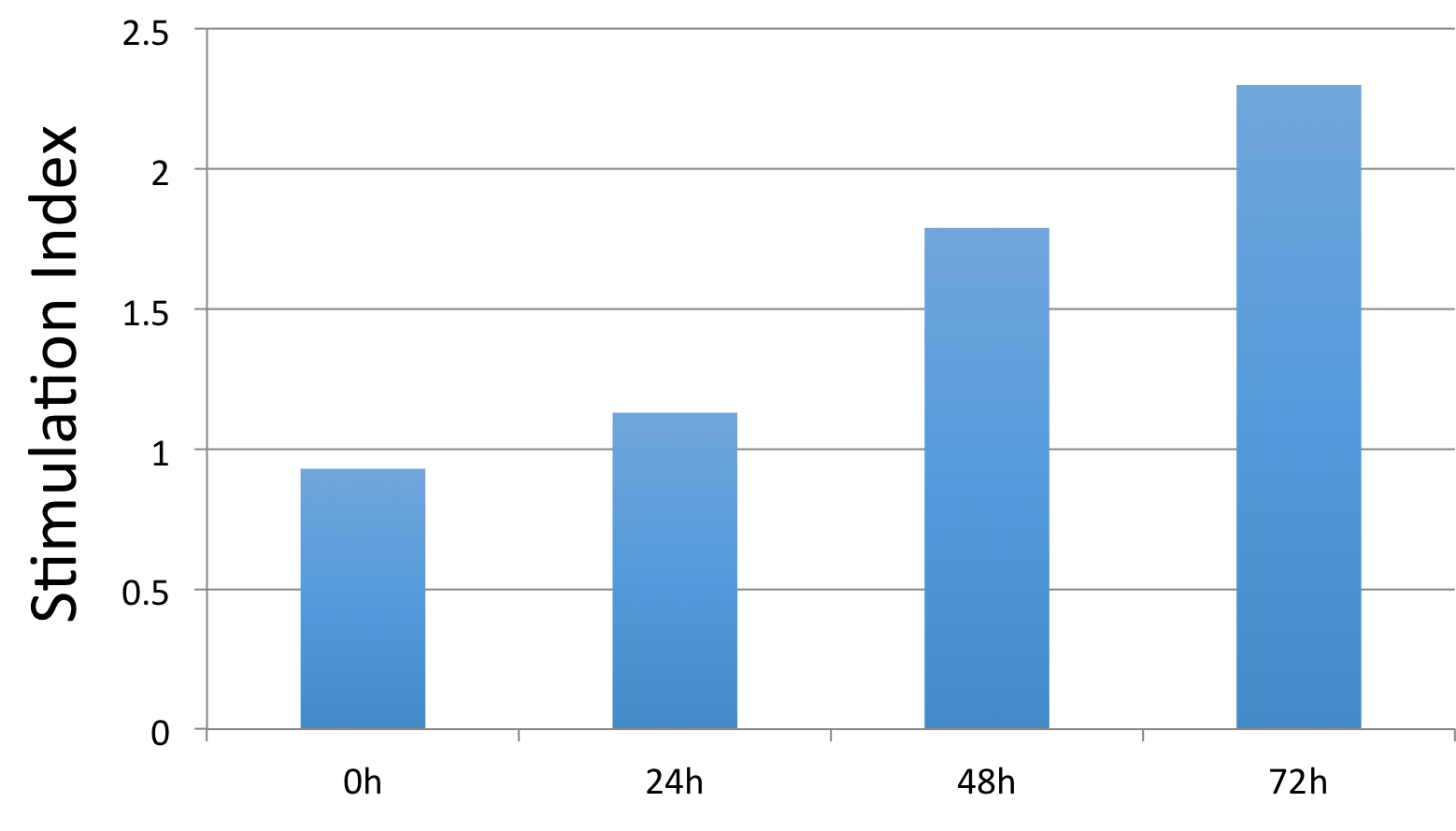


**Figure S2. p-STAT4 induction by IL-12 after various times of anti-CD3/CD28 activation in purified T-cells.**  Column-purified T-cells were activated with anti-CD3/CD28 beads for various times, then stimulated with 50 ng/ml IL-12 for 1 hour, fixed, permeabilized, and stained intracellularly for p-STAT4. Stimulation index = number of p-STAT4-positive cells after 1 hour of IL-12 stimulation divided by the corresponding value for the CD3/CD28-activated control. The results shown are from one of two experiments, which yielded very similar results.

**
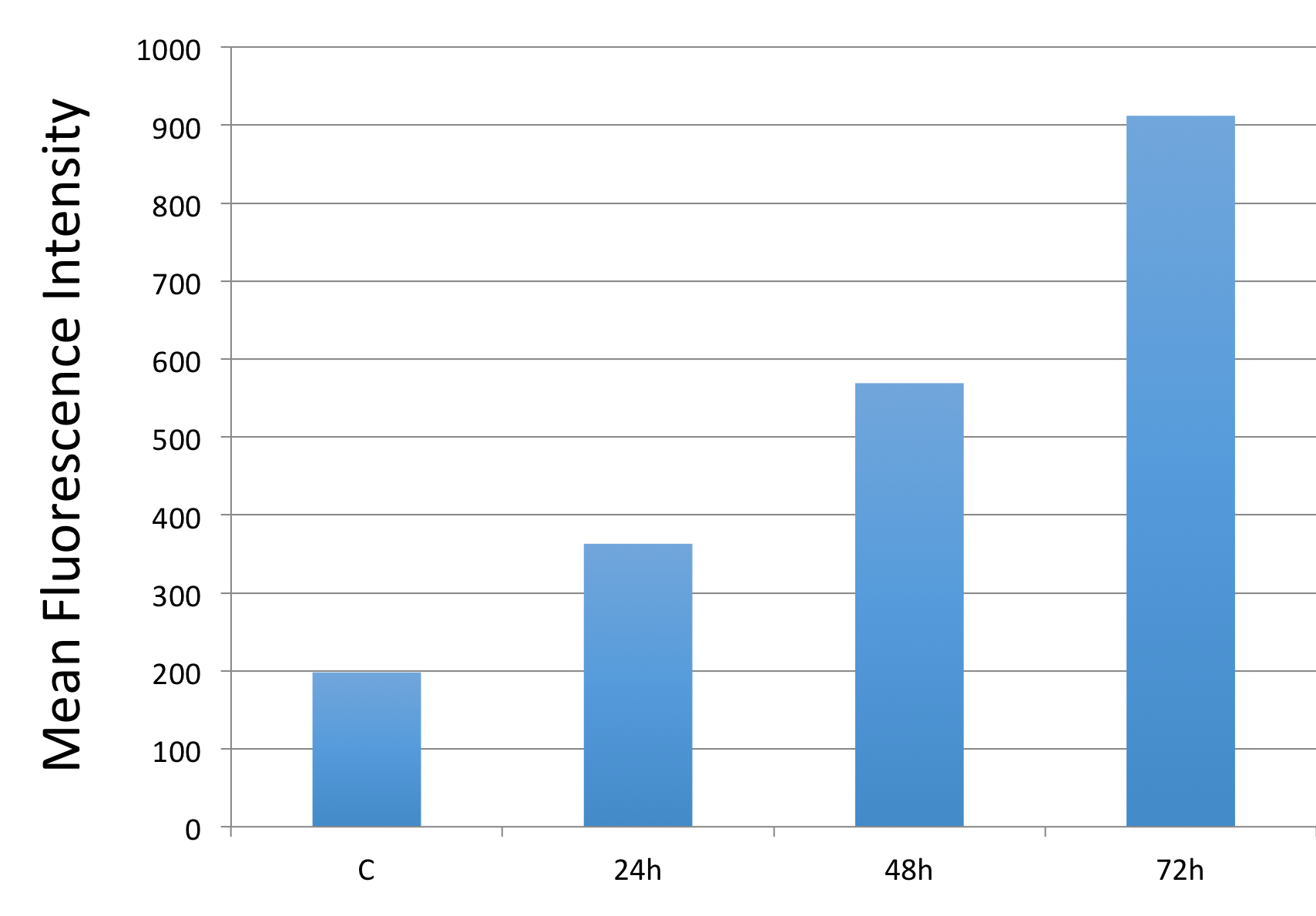
**

**Figure S3. Induction of cell surface IL-12RB1 after various times of anti-CD3/CD28 activation in purified T-cells.** Column-purified T-cells were stimulated with anti-CD3/CD28 beads for various times, followed by cell surface staining for IL12RB1. Mean Fluorescence Intensity is shown for the CD3+ T-cells. C = 72h, without beads. The data shown are from one of two experiments, which yielded very similar results.

**
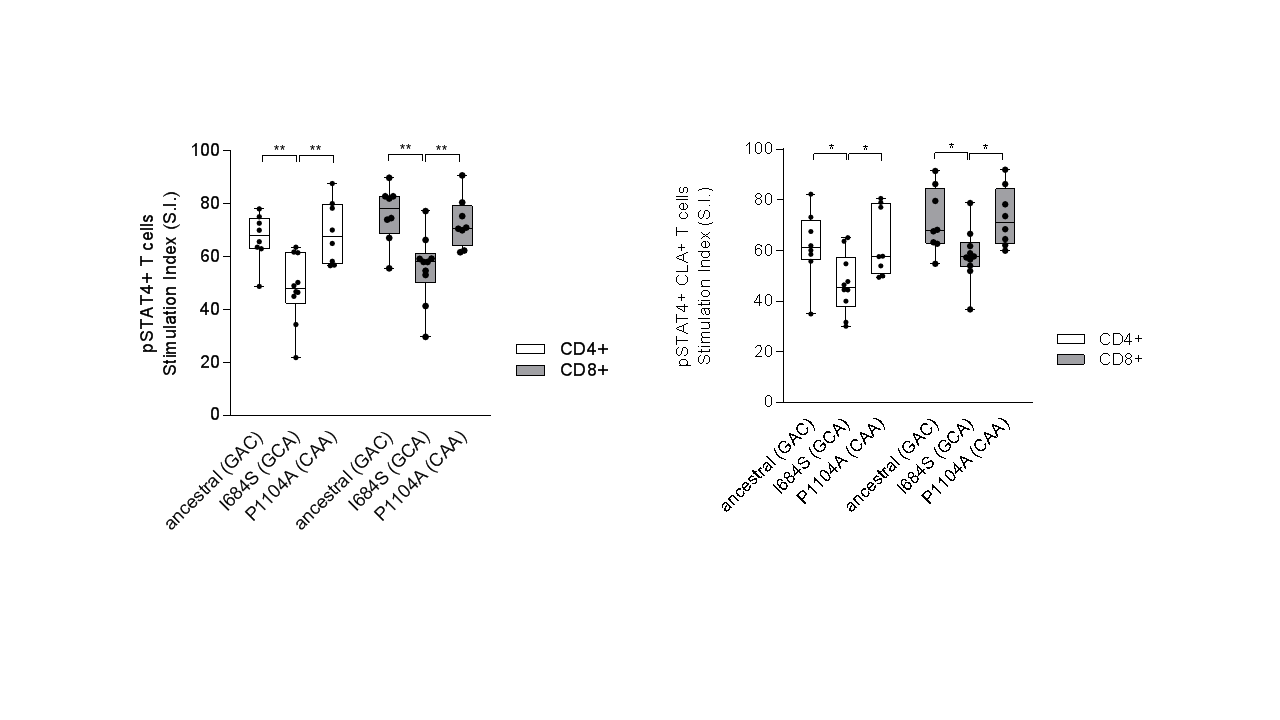
**

**Figure S4: Haplotype-related differences in IL-12 stimulated p-STAT4 persist after omitting psoriasis cases from the subject pool.**

Data from Figure 3 were re-analyzed to calculate the Stimulation Index (SI) for IL-12 stimulated p-STAT4, for (A) total CD4+ T-cells (open bars) and total CD8+ T-cells (gray bars), as well as (B) skin-homing CD4+CLA+ and CD8+CLA+ T-cells (open and gray bars, respectively). Box plots: midline indicates median, box indicates 25^th^ to 75^th^ percentile, and whiskers indicate minimum to maximum. * indicates p<0.05, ** indicates p<0.01, and *** indicates p<0.001.

**
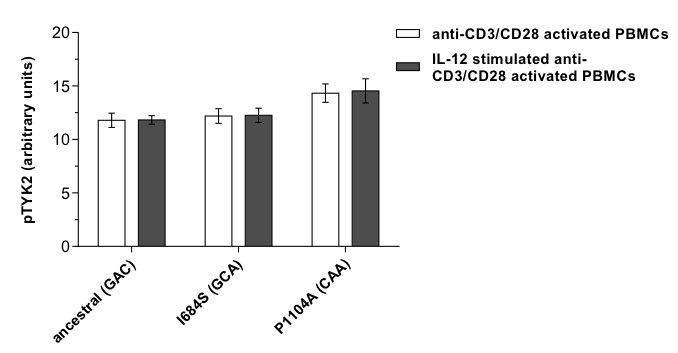
**

**Figure S5. Detection of phosphorylated TYK2 in cell lysates from anti-CD3/CD28 activated PBMCs with or without IL-12 stimulation.**

PBMCs derived from the ancestral haplotype (n=8) and from two haplotypes representing the genetic variants I684S (n=9) and P1104A (n=4), were cultured for 72h with anti-CD3/CD28 beads, rested overnight and then stimulated with IL-12 for 1h, lysed and analyzed for pTYK2 by ELISA. Gray bars display IL-12 stimulated anti-CD3/CD28 activated PBMCs and white bars display unstimulated activated PBMCs.

**References**

1 Nair, R. P. *et al.* Genome-wide scan reveals association of psoriasis with IL-23 and NF-kappaB pathways. *Nat Genet* **41**, 199-204, doi:ng.311 10.1038/ng.311 (2009).

2 Tsoi, L. C. *et al.* Identification of 15 new psoriasis susceptibility loci highlights the role of innate immunity. *Nat Genet* **44**, 1341-1348, doi:10.1038/ng.2467ng.2467 (2012).

3 Purcell, S. *et al.* PLINK: a tool set for whole-genome association and population-based linkage analyses. *Am J Hum Genet* **81**, 559-575, doi:S0002-9297(07)61352-410.1086/519795 (2007).

4 Tsoi, L. C. *et al.* Large-scale meta-analysis identifies 18 novel psoriasis susceptibility loci. *Nat Commun* **in press** (2017).

5 Purcell, S., Daly, M. J. & Sham, P. C. WHAP: haplotype-based association analysis. *Bioinformatics* **23**, 255-256, doi:10.1093/bioinformatics/btl580 (2007).

6 O'Connell, J. *et al.* A general approach for haplotype phasing across the full spectrum of relatedness. *PLoS Genet* **10**, e1004234, doi:10.1371/journal.pgen.1004234 (2014).

7 Trapnell, C. *et al.* Differential gene and transcript expression analysis of RNA-seq experiments with TopHat and Cufflinks. *Nat Protoc* **7**, 562-578 (2012).

8 Anders, S., Pyl, P. T. & Huber, W. HTSeq--a Python framework to work with high-throughput sequencing data. *Bioinformatics* **31**, 166-169, doi:10.1093/bioinformatics/btu638 (2015).

9 Love, M. I., Huber, W. & Anders, S. Moderated estimation of fold change and dispersion for RNA-seq data with DESeq2. *Genome Biol* **15**, 550, doi:10.1186/s13059-014-0550-8 (2014).

10 Tsoi, L. C. *et al.* Analysis of long non-coding RNAs highlights tissue-specific expression patterns and epigenetic profiles in normal and psoriatic skin. *Genome Biol* **16**, 24, doi:10.1186/s13059-014-0570-4 (2015).

11 Li, B. *et al.* Transcriptome analysis of psoriasis in a large case-control Sample: RNA-Seq rovides insights into disease mechanisms. *J Invest Dermatol* **134**, 1828-1838, doi:10.1038/jid.2014.28 (2014).
